# Sleep Quality and Self-Control: The Mediating Roles of Positive and Negative Affects

**DOI:** 10.3389/fpsyg.2020.607548

**Published:** 2020-12-17

**Authors:** Jinru Liu, Lin Zhu, Conghui Liu

**Affiliations:** Department of Psychology, Renmin University of China, Beijing, China

**Keywords:** sleep quality, self-regulation, positive affect, negative affect, mediation

## Abstract

This study examined the mediating roles of both positive and negative affects in the relationship between sleep quality and self-control. A sample of 1,507 Chinese adults (37% men; mean age = 32.5 years) completed self-report questionnaires measuring sleep quality, positive and negative emotions, and self-control. Poor sleep quality was positively correlated with negative affect and negatively correlated with positive affect and self-control. Positive affect was positively correlated with self-control, while negative affect was negatively correlated with self-control. Both positive and negative affects significantly mediated the relationship between sleep quality and self-control. Improving individuals’ sleep qualities may lead to more positive emotions and less negative emotion, and these mood changes may increase resources for self-control. Regulating positive and negative affects may reduce the negative effects of poor sleep quality on self-control.

## Introduction

Self-control is an important component of successful psychological functioning, and it alters one’s response when presented with conflicting desires (Pilcher et al., 2015). High trait self-control predicts low resting heart rate, high heart rate variability, a steep cortisol slope, stable emotional patterns, and well-being (Daly et al., 2012), which are beneficial for physical and psychological health. Self-control is also predictive of success in achievement-related domains (Choi et al., 2018). Self-control enables people to delay instant gratification and work toward long-term goals.

Neuroimaging indicates sleep deprivation affects activation and deactivation of the prefrontal cortex (PFC) (Chee and Choo, 2004), resulting in less-effective executive control function of the PFC (Nilsson et al., 2005). Poor sleep quality deleteriously impacts self-control capacity (Altena et al., 2008), such as emotion regulation (Palmer and Alfano, 2017) and cognitive inhibition (García et al., 2012). A diary study covering 10 working days reveals that a good night’s sleep allows employees to regain self-control the next day (Gombert et al., 2018). The underlying mechanism by which sleep quality impacts self-control, however, remains unclear.

Bouwmans et al. (2017) suggest that sleep quality can predict positive and negative affects. Negative affect was positively correlated with the Pittsburgh Sleep Quality Index (PSQI) score, and positive affect was negatively correlated with the PSQI score (Latif et al., 2019). Positive affect is related to better sleep quality (Bower et al., 2010). Disturbed sleep engenders lower positive affect and reduced psychological well-being (Steptoe et al., 2008). Subjective sleep quality is a predictor of next-day positive affect (de Wild-Hartmann et al., 2013), which is consistent with an electronic diary study (Bouwmans et al., 2017). Poorer sleep is predictive of elevated levels of negative affect (Sin et al., 2017; Shen et al., 2018), and better sleep is predictive of increased positive affect (Hamilton et al., 2008; Kalmbach et al., 2014).

Sleep duration is not exactly the same as sleep quality, but it is usually one of the components included in measures of sleep quality, such as the PSQI. Some relevant studies have shown that individuals’ sleep durations are also correlated with positive and negative affects. Franzen et al. (2008) found that experimental sleep deprivation results in reduced positive mood and increased negative mood. Sleep loss is associated with increased negative affective responses to stressors, and sleep deprivation reduces individuals’ psychological thresholds for the perception of stress from cognitive demands. One study using cross-sectional data shows that insomnia was associated with negative affect, which in turn was associated with marijuana-related problems (Yurasek et al., 2020). The protective function of good sleep quality was verified by Sonnentag and Binnewies (2013) – sleep quality buffers the positive effects of negative affect during the evening on negative affect the next morning. Lack of sleep also hinders emotion regulation (Baumeister and Heatherton, 1996), whereas adequate sleep promotes self-control and emotion regulation (Zohar et al., 2005; Hamilton et al., 2008).

Negative affect may induce self-control failure (Heatherton and Wagner, 2011). Binge eating, for example, is associated with loss of self-control and is adopted to avoid negative affect (Mason et al., 2018). Anger is a common negative affect, which exerts a prominent effect on aggressive behaviors and hinders individuals’ self-control (Ellwanger and Pratt, 2014). Negative affect is also associated with risky decision-making, irrational consumption (Bruyneel et al., 2009), and addictive behaviors (Wilson et al., 2014). Individuals with poor sleep quality may act more impulsively to cope with the associated negative mood, presenting as the decrease of individual self-control ability (Zhu et al., 2019).

Self-control was regarded as a limited resource in the self-control strength model (Tice et al., 2007), and positive affect compensates the depletion of self-control and facilitates self-control behaviors (Shmueli and Prochaska, 2012). Individuals with high (vs. low) positive affect are likely to consider situational details, act accordingly, and stay on uninteresting tasks longer (Isen and Reeve, 2005).

Although some have examined the mediating role of positive affect in the association between sleep quality and drug craving (Freeman and Gottfredson, 2018), few studies simultaneously investigated the mediating roles of positive and negative affects in the relationship between sleep quality and self-control. Therefore, we tested a two-mediator model assuming that sleep quality predicts self-control and that positive and negative affects mediate this association.

## Materials and Methods

### Participants

A total of 1,507 participants (37% men; age range = 18–59 years; mean age = 32.5 years) received a small reward (10¥≈1.49$) as compensation for participation in the study. Participants who were working in various industries and companies (e.g., governmental agencies and institutions, nationalized business, foreign companies, and private companies) in Beijing participated. To ensure heterogeneity, we collected data from participants of different ages, education levels, marital statuses, income levels, and parental situations from different urban areas in Beijing.

### Measures

#### Sleep Quality

The Chinese version of the PSQI was applied to measure participants’ sleep quality (Liu et al., 1996). The scale was designed (Buysse et al., 1989) to measure seven components of sleep quality: subjective sleep quality, sleep latency, sleep duration, habitual sleep efficiency, sleep disturbances, use of sleeping medication, and daytime dysfunction over the last month. The scale comprises 19 items (e.g., “During the past month, when have you usually gone to bed?”) and each component has a score ranging from 0 to 3, with higher total scores indicating poorer sleep quality. Cronbach’s α for the Chinese version of components of the PSQI in this study was 0.76.

#### Positive and Negative Affects

Positive and negative affects were measured by the Chinese version (Huang et al., 2003) of the Positive and Negative Affect Schedule, which was designed by Watson et al. (1988) to evaluate the tendency to experience positive (10 items; e.g., “enthusiastic”) and negative (10 items; e.g., “nervous”) affect. The scale comprises 20 items, and each item was rated on a Likert scale ranging from 1 (*not at all*) to 5 (*extremely*). Cronbach’s αs for positive and negative affects were 0.86 and 0.87, respectively, in this study.

#### Self-Control

The Chinese version of the Self-Control Scale (Tan and Guo, 2008) was adopted to measure participants’ self-control, which was designed by Tangney et al. (2004). In this study, the scale comprised 19 items to evaluate five components of self-control: resisting temptation, impulse control, healthy habits, task performance, and entertainment temperance (e.g., “I am good at resisting temptation”). Answers were provided on a Likert scale ranging from 1 (*not at all)* to 5 (*very much*), with higher total score signifying better self-control. Cronbach’s α for the Chinese version of self-control in this study was 0.88.

### Procedure

The questionnaires were uploaded onto Wen Juan Xing, an online platform used to collect survey data. After the questionnaire link was generated on this platform, we spread the questionnaire link through WeChat (an instant messaging software) Moments. Participants were recruited through WeChat Moments, and standardized instructions were employed to direct participants on how to complete the questionnaires. Participants completed a survey designed that comprised demographic variables, sleep quality, positive and negative affects, and self-control. Sixty participants were excluded due to incomplete answers. The time window of data collection was from August 2018 to October 2018. Informed consent was obtained from all participants, and this study was approved by the scientific ethical committee of the authors’ university.

## Results

### Preliminary Analyses

Analyses were conducted in IBM SPSS Statistics 24. Data were screened for missing values, normality, and selection bias prior to analysis. Means, standard deviations, and zero-order correlations for all study variables are shown in Table 1. The PSQI score was positively associated with negative affect (*r* = 0.26, *p* < 0.01), and negatively associated with positive affect (*r* = −0.21, *p* < 0.01) and self-control (*r* = −0.25, *p* < 0.01), indicating that people with better sleep quality tend to experience more positive affect and self-control and less negative affect. Self-control was negatively correlated with negative affect (*r* = −0.49, *p* < 0.01) but positively correlated with positive affect (*r* = 0.24, *p* < 0.01), elucidating that both sleep quality and positive affect are protective factors for self-control (Table 1 about here).

**TABLE 1:** Descriptive statistics and correlations between the variables (*N* = 1507).

**Variable**	***M***	***SD***	**1**	**2**	**3**	**4**
1. Positive affect	3.34	0.65	–			
2. Negative affect	2.60	0.70	0.10**	–		
3. Self-control	3.21	0.59	0.24**	−0.49**	–	
4. PSQI Score	5.66	3.01	−0.21**	0.26**	−0.25**	–

**p < 0.01.

### Analyses of the Mediating Roles of Positive and Negative Affects

This study assumed that positive and negative affects would mediate the relationship between sleep quality and self-control. We utilized Model 4 from PROCESS 3.0 module in SPSS (Hayes, 2013) to acquire and compare the mediating effects of positive and negative affects. To elucidate the estimated model in PROCESS, 5,000 bootstrap samples were used to estimate the indirect effect using the bias-corrected bootstrap confidence interval.

As presented in Table 2, three regression models with positive affect, negative affect, and self-control as criteria were estimated, respectively. The mediation analysis indicated that with self-control regressing onto all the predictor variables, positive affect imposed a positive effect on self-control, and negative affect imposed a negative effect on self-control. Bootstrapped 95% confidence intervals (CIs) verified that the indirect effects of positive and negative affects in the relationship between sleep quality and self-control were significant (Table 2 about here).

**TABLE 2:** Testing the two-mediator model using PROCESS (*N* = 1507).

**Antecedent variable**	**Consequent variable**					
	**Negative affect**		**Positive affect**		**Self-control**	
	**β**	***t***	**β**	***t***	**β**	***t***
PSQI Score	0.06	10.41***	−0.05	−8.29***	−0.01	−2.51*
Positive affect	–	–	–	–	0.25	12.64***
Negative affect	–	–	–	–	−0.42	−22.55***
Constant	2.25	60.75***	3.60	102.50***	3.53	43.80***
*F*	108.32***		68.79***		241.94***	
*R*^2^	0.07		0.04		0.33	

*p < 0.05, ***p < 0.001.

**FIGURE 1:**
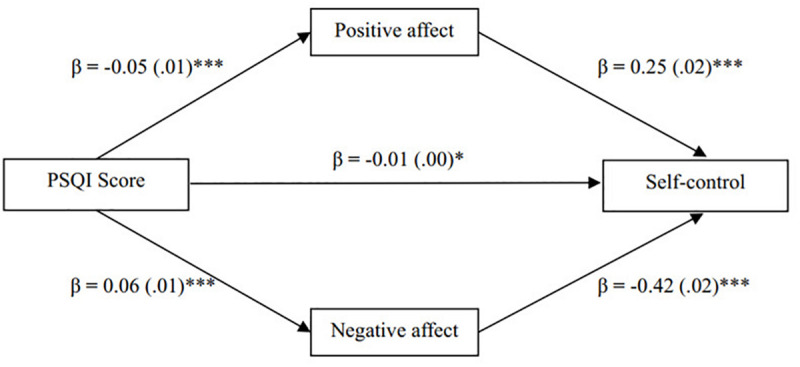
The two-mediator model on the relation of sleep quality to self-control *p < 0.05, ***p < 0.001.

Figure 1 presents the results of the mediation model concerning the effects of sleep quality on self-control through positive and negative affects. The PSQI score, positive and negative affects accounted for approximately 33% of the variance in self-control [*R*^2^ = 0.33, *F*(3,1503) = 241.94, *p* < 0.001]. There was a significant negative association between PSQI score and positive affect (β = −0.05, *SE* = 0.01, *p* < 0.001), suggesting that greater sleep quality was associated with more positive affect, and positive affect significantly predicted self-control (β = 0.25, *SE* = 0.02, *p* < 0.001) (Figure 1 about here). Conversely, there was significant positive association between the PSQI score and negative affect (β = 0.06, *SE* = 0.01, *p* < 0.001), suggesting that poorer sleep quality was associated with more negative affect, and negative affect significantly predicted self-control (β = −0.42, *SE* = 0.02, *p* < 0.001).

An indirect effect is significant when the 95% bootstrap CI does not include zero (Hayes, 2013). According to this principle, the total effect of sleep quality on self-control (*effect* = −0.05, *t* = 9.78, *p* < 0.001) was separated into the direct effect (*effect* = −0.01, *t* = 2.51, *p* < 0.05), and the two indirect effects *via* positive (*indirect effect* = −0.01, 95% CI = [−0.02, −0.01]) and negative (*indirect effect* = −0.03, 95% CI = [−0.03, −0.02]) affect, respectively. Therefore, positive and negative affects mediated the relationship between sleep quality and self-control. Comparing the mediating effects of positive and negative affects, there was significant difference between the two indirect effects (*effect difference* = 0.01, 95% CI = [0.01, 0.02]). The total model (*R*^2^ = 0.33, *F* = 241.94, *p* < 0.001) accounted for 33% of the variance in self-control.

## Discussion

The purpose of this study was to investigate the mediating roles of positive and negative affects in the relationship between sleep quality and self-control. We tested a two-mediator model hypothesizing that sleep quality predicts self-control and that positive and negative affects mediate this association. Consistent with a mini-review that explored the intersection between sleep habits and self-control (Pilcher et al., 2015), a negative association between the PSQI score and self-control was found in this study, and the relationship between sleep quality and self-control was mediated by positive and negative affects differently.

A two-mediator model was employed to explore the relationship among sleep quality, affect, and self-control. There are two paths through which sleep quality impacts self-control: one path is that poor sleep quality might increase negative affect and decrease self-control ability. The first stage is consistent with links between sleep quality and negative affect (Shen et al., 2018), and also in line with a recent clinical observational study (Reeve et al., 2018). Sleep deprivation results in increased negative mood (Franzen et al., 2008), and poorer sleep quality elevates negative affect (Sin et al., 2017; Shen et al., 2018).

To avoid negative affect, people may employ maladaptive behaviors, which is related to loss of self-control (Mason et al., 2018). For these people, their inhibitory functions are used inefficiently and excessively, which might result in eventual self-regulatory fatigue (Baumeister and Vohs, 2010; Chester et al., 2016). A neuroimaging study explained the association between negative affect and self-control – negative emotions lead to self-control failure owing to their excessively recruitment of inhibitory regions of the PFC (Chester et al., 2016). Excessive reactivity of the brain’s regulatory resources results in the failure of self-control (Chester et al., 2016). Sleep loss and poor sleep quality may adversely affect individuals’ abilities to cope with stressful events, which increases the likelihood of self-control failure. Sleep duration is one of the dimensions to measure sleep quality by the PSQI, and researchers have verified that a lack of sleep affects the ability to control emotions (Baumeister and Heatherton, 1996); according to the self-control strength model (Tice et al., 2007), emotion regulation occupies the general resources supporting self-control. While sleep can be used as the recovery resource of self-control, adequate sleep enables one with more self-control energy for emotion regulation (Zohar et al., 2005; Hamilton et al., 2008).

The other path of the model is that better sleep quality might increase positive affect and decrease failure of self-control. Sufficient sleep and fewer sleep disorders are predictive of positive affect (Sin et al., 2017). According to the model of conservation of resources, sleep promotes the recovery of positive affect (Hobfoll, 1989). Furthermore, positive affect enables individuals to more effectively exercise self-control. The broaden-and-build theory of positive affect can be employed as an interpretation – positive emotion can broaden attention scope and improve cognitive flexibility (Fredrickson, 2004). The effects of positive emotions are cumulative, indirect, and long-term, which will be stocked and further contribute to construction of individual resources. Related studies reflect that positive affect helps improve self-regulation in the case of ego depletion (Tice et al., 2007).

From the perspective of emotion regulation, the relationship between sleep quality and self-control can be further explained. Previous studies have shown that poor sleep quality may impair the ability of cognitive reappraisal and then lead to lower ability to regulate negative affect (Mauss et al., 2013). A study adopting functional magnetic resonance image supported this (Yoo et al., 2007). The medial PFC (MPFC) has a controlling function, and the amygdala modulates emotional responses. Compared to participants with better sleep quality, the link between the amygdala and MPFC was weaker in sleep-deprived participants.

According to the Self-Control Strength Model (Tice et al., 2007), negative affect is problematic because it prompts the need for emotion regulation, which draws from a limited pool of domain-general resources supporting self-control (Baumeister and Heatherton, 1996). In this model, self-control is a limited resource, which is depleted after performing self-control tasks (Muraven and Baumeister, 2000). Positive affect counteracts the depletion of self-control and facilitates self-control behaviors (Shmueli and Prochaska, 2012).

One possible mechanism by which sleep quality may impact self-control was revealed in this study. We examined the mediating roles of positive and negative affects simultaneously and found that both positive and negative affects mediate the relationship between sleep quality and self-control. Adequate sleep and better sleep quality can not only increase positive emotions but also reduce negative emotions, and both play important roles in promoting self-control. Sleep quality showed an association with individuals’ positive and negative affects, which are closely related to the success or failure of self-control and further affects individuals’ physical and mental health. The ratio of the total indirect effects to the total effect reached 80%, highlighting the importance of both positive and negative affects as mediators in transmitting the impact of sleep quality to self-control.

This study also had some limitations. First, the measurement mainly relies on the self-report method, which may be affected by social desirability and self-serving bias (Dunning et al., 1989, 1991; Lindeman and Verkasalo, 1995). In addition, the PSQI is an effective way to measure sleep quality; however, participants may develop false memories of sleep quality because of its retrospective nature. Further studies should employ more timely measures of sleep quality; for example, ecological momentary assessment, which is characterized by repeated measurements of how participants feel in real situations. Researchers could assess sleep quality on a daily basis by using a “sleep diary” approach, so that each morning the person rates his/her sleep quality on the night before (Eerde and Venus, 2018). Second, the cross-sectional data were collected and correlation analyses were conducted, which means we cannot make causal inferences. Experimental methods and longitudinal data can be used to clarify the causal relationships between variables in the future. Third, in this study, 63% of the participants were women and 37% were men, which shows a relatively imbalanced gender proportion. Future studies should maintain a relatively balanced sex ratio of participants. Lastly, in this model, some variables that have key influences on self-control were not considered, such as the role of emotion regulation. In the future, emotion regulation can be included to supplement the model.

Despite these limitations, this study reveals an important finding that better sleep quality might not only directly lead to higher levels of self-control but also improve level of self-control by reducing negative affect and increasing positive affect. This result both deepens our understanding of the relationship between sleep and self-control and provides a new perspective for increasing individual self-control. Our study has practical implications for individuals with self-control disorders; our results suggest that poorer sleep quality is associated with more negative affect, and that individuals with poorer sleep quality adopt impulsive behaviors in response to negative emotions. In addition, our findings have clinical implications for individuals with substance abuse. Individuals with substance abuse may benefit from intervention programs that target negative affect and sleep. For non-clinical populations, our findings suggest that increasing sleep quality might improve human emotions, thereby enhancing their self-control ability. Developing good habits of sleeping and regulating emotions could prevent individuals from making impulsive decisions. The present findings support further research investigating whether intervention programs of improving sleep quality and mood can increase the ability of clinical and non-clinical groups to implement self-control.

## Data Availability Statement

The raw data supporting the conclusions of this article will be made available by the authors, without undue reservation.

## Ethics Statement

The studies involving human participants were reviewed and approved by the Academic Ethics Committee, Department of Psychology, Renmin University of China. Written informed consent for participation was not required for this study in accordance with the national legislation and the institutional requirements.

## Author Contributions

CL designed the original study and conducted final critical revision. JL conducted literature searches and statistical analysis. JL and LZ wrote the manuscript. All authors contributed to the article and approved the submitted version.

## Conflict of Interest

The authors declare that the research was conducted in the absence of any commercial or financial relationships that could be construed as a potential conflict of interest.
